# *Gattaca* as a lens on contemporary genetics: marking 25 years into the film’s “not-too-distant” future

**DOI:** 10.1093/genetics/iyac142

**Published:** 2022-10-11

**Authors:** C Brandon Ogbunugafor, Michael D Edge

**Affiliations:** Department of Ecology and Evolutionary Biology, Yale University, New Haven, CT 06520, USA; Santa Fe Institute, Santa Fe, NM 87501, USA; Vermont Complex Systems Center, Burlington, VT 05401, USA; Department of Quantitative and Computational Biology, University of Southern California, Los Angeles, CA 90089, USA

**Keywords:** embryo selection, forensic genetics, genetic discrimination, heritability, polygenic scores, science and society, science fiction

## Abstract

The 1997 film *Gattaca* has emerged as a canonical pop culture reference used to discuss modern controversies in genetics and bioethics. It appeared in theaters a few years prior to the announcement of the “completion” of the human genome (2000), as the science of human genetics was developing a renewed sense of its social implications. The story is set in a near-future world in which parents can, with technological assistance, influence the genetic composition of their offspring on the basis of predicted life outcomes. The current moment—25 years after the film’s release—offers an opportunity to reflect on where society currently stands with respect to the ideas explored in *Gattaca*. Here, we review and discuss several active areas of genetic research—genetic prediction, embryo selection, forensic genetics, and others—that interface directly with scenes and concepts in the film. On its silver anniversary, we argue that *Gattaca* remains an important reflection of society’s expectations and fears with respect to the ways that genetic science has manifested in the real world. In accompanying supplemental material, we offer some thought questions to guide group discussions inside and outside of the classroom.

## Teaser

In this article, Ogbunugafor and Edge commemorate the 25th anniversary of the release of the film *Gattaca*, using the moment as an opportunity to examine contemporary issues—both technical and ethical—in modern human genetics.

##  

The 1997 film *Gattaca*, written and directed by Andrew Niccol, envisions a “not-too-distant” future in which predictions made on the basis of genotypes play a central role in structuring society. Despite positive reviews from critics, *Gattaca* underperformed at the box office in its initial theatrical run. Since then, however, *Gattaca* has become a cult classic, and its title is a stand-in for the idea of genetic dystopia in popular parlance (e.g. Regalado 2019).

*Gattaca*’s protagonist is Vincent Anton Freeman (Ethan Hawke), who was conceived the old-fashioned way, without the aid of modern technology. So-called “faith births” have become taboo in *Gattaca*’s society—such people are labeled as “in-valids,” “*uteros*,” or “God children” and barred from opportunities and status. Based on what is in his genome, Vincent is predicted at birth to be at risk for several conditions, including a heart disorder foretold to lead to early death, which precludes his participation in educational and social institutions and in most employment opportunities.

Because of this genetic discrimination—outlawed in principle but common in practice in *Gattaca*’s world—doors are closed to Vincent that would allow him to pursue his lifelong dream of becoming an astronaut at the Gattaca Aerospace Corporation. This goal is unreachable until he takes bold and desperate action, adopting the genetic identity of another person: an illegal, underground process called a “borrowed ladder.” Vincent pays to adopt the genetic persona of a cooperating “valid”—that is, a person born under genetic supervision and assistance—Jerome (Jude Law), a former elite swimmer whose paraplegia prevents him from competing.^1^ Jerome’s genetics give Vincent access to a job at the Gattaca Aerospace Corporation. Early in the film, a murder at the Gattaca Corporation jeopardizes Vincent’s upcoming mission to Titan, and the ensuing investigation threatens to reveal his true identity.

Used widely in classrooms after its release, *Gattaca* remains as relevant as ever, a lens on modern uses of genetics. A quarter century later, we look back on the film, considering it from our perspective as geneticists. We organize our discussion around major scenes in the movie, taking up its depiction of genetic discrimination, genetic prediction, embryo selection, genetic investigations of crime, gene-by-environment interactions, and related topics. Along the way, we briefly review some technical aspects of contemporary genetics work related to *Gattaca*. For clarity, we have provided a brief definition of these concepts (Supplementary Table 1) and a list of the main characters in the film (Supplementary Table 2). We also discuss how the ideas in *Gattaca* have appeared in analogous social contexts in the United States. In Supplementary Text, we suggest questions for guiding classroom and community discussions of *Gattaca*. *Caveat lector*, minor spoilers await.

## “We now have discrimination down to a science”

Though *Gattaca* has acquired a status as the quintessential genetics movie, the film’s central messages are about discrimination writ large. The film makes the point that discrimination occurs along any of a number of dimensions (e.g. race, religion, gender identity), with genetics the instrument of discrimination in this fictional world. The science of genetics has already been a close partner of discrimination in its history, including via the eugenics movement (Kevles 1995). *Gattaca* imagines a reinvigorated partnership in an era of enhanced genetic technology.

Vincent speaks to this in the film’s opening scene, describing his plight via narration: “I belonged to a new underclass, no longer determined by social status or the color of your skin. No, we now have discrimination down to a science.” *Gattaca* depicts a world organized according to genetic predictions rather than the realized outcomes of genetic variation. For Vincent, his status as an in-valid, coming with predictions of elevated disease risk and limited ability, excludes him from much of society. For Jerome, laden with genetic gifts, the weight of prophecy is also crushing, leading to a wrenching personal crisis after he falls short of expectations.

The world of *Gattaca* is one in which the most privileged (valids) can propagate their privilege by ensuring that their children are born with genetic assistance. Privilege also becomes naturalized, justified by scientific prediction. Economic barriers to genetic assistance undercut Vincent’s narrative claim that class is “no longer determined by social status.” Through his experiences, Vincent realizes that though genetic variation matters, the social force of genetic prediction can be at least as powerful. Uncertainty about whether Vincent will continue to outwit the genetically defined elite, in part by capitalizing on their misplaced faith in an overstated genetic determinism, is an important driver of tension in the film’s plot.

## “There’s no gene for fate”

Predictions are powerful in *Gattaca* despite their uneven performance. Some commentators have taken the overly precise predictions in *Gattaca* at face value. For example, futurist Ramez Naam wrote (Naam 2014), “…the science in the film was just terrible. No genetic test will ever tell you how many heartbeats you have left…The film was a gross exaggeration.” Naam is correct that the precision purported to be obtained by some of the predictions in *Gattaca* is impossible. Our reading is that the film acknowledges this and constantly undercuts the presumption that the predictions work as the characters seem to expect, suggesting thereby that overstatements of the predictive power of genetics are a powerful social tool.

Early in *Gattaca*, we see a flashback to the birth of the main character, Vincent. During Vincent’s first moments, one member of the medical team recites predictions about Vincent’s future from a monitor:

“Neurological condition: 60% probability

Manic depression: 42% probability

Attention deficit disorder: 89% probability

Heart disorder: 99% probability

Early fatal potential, life expectancy: 30.2 years”

Vincent narrates, “My destiny was mapped out before me—all my flaws, predispositions, and susceptibilities—most untreatable to this day. Only minutes old, the date and cause of my death was already known.”

The events of the film cast the value of the fortune being told into doubt. Vincent, after all, has already outlived his predicted demise from heart failure at the time of the story^2^ and does not, to the viewer’s knowledge, develop most of the diseases predicted at his birth.^3^ Foretold to be frail, Vincent becomes a strong athlete, eventually outswimming his genetically assisted brother, Anton. Although Vincent is predicted to have a substantial chance of developing a mood disorder, it is Jerome, a valid, who struggles with depression.

The film does give the impression that genetic prediction has proven accurate and precise for morphological traits, in fact, more precise than would seem to be possible on the basis of current estimates of environmental contributions to such traits (Polderman *et al.* 2015). The record of genetic prediction in *Gattaca* appears, but is not stated, to be shakier in other areas, including disease, performance, and behavior. That said, the predictions being made are probabilistic, and one person failing to develop a condition predicted to develop with probability <1 does not by itself invalidate the prediction method, which may be accurate at a population level. Even if the accuracy of the predictions made in *Gattaca* is presumed, however, most characters in the movie appear to treat them as essentially deterministic, though they are not.

Prediction of traits has been a central pursuit of genetics for decades. In agriculture, genomic prediction (Meuwissen *et al.* 2001) has been broadly useful (Crossa *et al.* 2017; Georges *et al.* 2019) and transformative in some cases, for example increasing the rate of improvement from breeding programs of milk yield in dairy cattle by about twofold (Wiggans *et al.* 2017).^4^ There has also been excitement about the value of genetic predictions in human health, but there are important differences between agricultural and human health applications, including the possibility of controlled breeding and environments in agriculture, and also distinct goals—individual prediction in the human case and shifting of a distribution in the agriculture case. Though rough predictions of disease risk could potentially be useful for screening purposes, accurate, personalized predictions of disease risk or response to treatment could transform healthcare. However, for many of the most medically important traits, such predictions have been slower to arrive than some might have expected.

For Mendelian diseases—diseases whose development is controlled by a person’s genotype at a single genetic location—prediction can be very accurate. Nevertheless, there can be considerable variation in how classically Mendelian diseases (e.g. sickle cell disease) manifest, as they can be modified environmentally (Piel *et al.* 2017; Royal *et al.* 2021), and there can also be uncertainty about uncharacterized variants in genetic loci known to generate Mendelian disease. Many other diseases, including most common forms of heart disease and cancer, are “complex” diseases, meaning that they are affected by numerous genetic variants, environmental factors, and their interaction. Most complex traits that have been studied appear to be extremely polygenic, with associated variants scattered across much of the genome (Boyle *et al.* 2017; Visscher *et al.* 2017; Sella and Barton 2019).

Box 1. “My destiny was mapped out before me”

The liability threshold model (Dempster and Lerner 1950; Falconer 1965) is the most popular framework for linking multilocus genotypes to disease risk. (See Supplementary Text for more background.) The liability threshold framework allows us to get a sense of how unusual the predictions recited at Vincent’s birth might be. To do this, we imagine that the liability threshold model holds exactly; that, in the world of *Gattaca*, healthcare providers have access to either exact liabilities or exact liabilities accessible via common SNPs; and that heritabilities and disease prevalences are similar to those measured in Western societies in the 20th century.

Given the liability threshold model, we can compute the percentile on the genetic liability scale necessary to achieve a given disease risk. The expression, justified in Supplementary Text, is

(1) $$\Phi ([\Phi^{-1}(1-p)-\sigma_{E}\Phi^{-1}(1-r)]\;/\;\sigma_{G}),$$

where Φ is the cumulative distribution function of the standard normal distribution, *p* is the disease prevalence, *r* is the predicted individual disease risk, and *σ_E_* and *σ_G_* are the standard deviations of the environmental and genetic components of liability, respectively.

The closest analog of “manic depression” is bipolar I disorder, which has an estimated worldwide prevalence of ≈1%, liability-scale SNP heritability (i.e. heritability explained by common variants) of ≈20%, and estimated (from twin studies) heritability of ≈80% in Western samples of European ancestries (O’Connell and Coombes 2021). Plugging these numbers into Equation (2) implies that to receive Vincent’s risk estimate of 42%, a person would need to be at the 99.3th percentile of genetic liability, or, if liability is computed from common SNPs, the 99.9999th percentile. Similarly, for “Attention Deficit Disorder,” the closest analog is Attention Deficit Hyperactivity Disorder, with estimated prevalence ≈5%, liability-scale heritability ≈74%, and liability-scale SNP heritability ≈18% in Western samples of European ancestries (Faraone and Larsson 2019). Under the liability threshold model, Vincent’s genetic risk estimate of 89% is thus at the 99.6th percentile of genetic risk if the full liability is known, or the 99.999999995th percentile if the liability is computed from common variants.

Under the assumptions used here, the risk predictions given to Vincent would place him in the tail of genetic risk for these conditions. To say how improbable this set of predictions would be for a random person, we would need to make assumptions about how the risks were selected for reporting (is it the same set for every birth, or are only high risks noted?), as well as about the correlations of genetic liability for the diseases considered.

One question raised by the probabilities recited at Vincent’s birth (considered more technically in Box 1) is the level of confidence we might ever conceivably place in the prediction of a complex phenotype. The environmental influences on complex traits mean that genetic near-identity is not enough to ensure identical outcomes (Wong *et al.* 2005; Roberts *et al.* 2012). For example, among people with bipolar I disorder (the closest contemporary analog to the “manic depression” of *Gattaca*’s screenplay) who have an identical twin, the twin has bipolar I disorder only about 40% of the time (Barnett and Smoller 2009).

The potential to predict a trait from genetic information is limited by the trait’s heritability, the proportion of trait variance explained by genetic variation in a given population and range of environments. Heritability can be estimated in many ways (Visscher *et al.* 2008; Zaitlen and Kraft 2012; Brandes *et al.* 2022).

For most human traits, genetic prediction falls well short of the upper limit predicted by heritability estimates, despite the massive scale of studies devoted to estimating the associations between genetic variation and trait variation, which now commonly include hundreds of thousands of research participants.

For the complex trait that has been most intensively studied, human height, a titanic meta-analysis of 5.4 million people has led to a genetic predictor—i.e. a “polygenic score” (see Box 2 for background) (Torkamani *et al.* 2018; Thompson *et al.* 2022)—that explains 40% of the variance in height in a sample of people of European ancestries (Yengo *et al.* 2022). This enormous effort achieved an accuracy approaching the “SNP heritability” (Yang *et al.* 2017; Hou *et al.* 2019), which is the portion of the heritability explained by common genetic variants. The SNP heritability is likely an upper bound on the performance of genetic prediction for the foreseeable future (Wray *et al.* 2021). For height, the heritability estimated from twin studies is usually estimated at about double the SNP heritability, around 80% (Visscher *et al.* 2008). Indeed, for many traits, the SNP heritability is substantially lower than heritability estimated from twin studies, but the relative importance of several nonexclusive explanations is not clear (Young 2019).

Modest SNP heritabilities delay the prospect of predictions for complex traits with precision and accuracy levels approaching those advertised in *Gattaca*, even in cases in which such precision could be possible in principle. At the same time, generalizing genetic predictions of complex traits outside the settings in which they have been calibrated has proven difficult. For example, predictions from the massive height study mentioned above accounted for only 10–20% of the variance in height outside people of European ancestries (Yengo *et al.* 2022), a pattern that has been observed repeatedly in analogous contexts (Martin *et al.* 2017, 2019; Thompson *et al.* 2022). The causes of these problems with generalizability are not fully understood, but evidence points to contributions from worldwide differences in the frequencies and correlations of genetic variants (Wang *et al.* 2020) as well as genetic interactions (Mostafavi *et al.* 2020; Patel *et al.* 2022; Zhu *et al.* 2022), in which the effect of a genetic variant on a trait depends on the environment (gene-by-environment interaction) or on genotypes at other positions in the genome (gene-by-gene interaction, or epistasis).

Despite such challenges, polygenic scores may prove valuable in the clinic, and for specific purposes and conditions, including coronary artery disease, they already show promise (Khera *et al.* 2018), especially in combination with known pathogenic variants (Maamari *et al.* 2022).

Alongside whatever clinical usefulness they provide, genetic predictions raise ethical and legal challenges, including genetic discrimination (Joly *et al.* 2020). In the United States, employers and health insurance providers are barred from considering genetic information under the Genetic Information Nondiscrimination Act (GINA). GINA, however, does not cover life, long-term care, or disability insurance, and at least one woman has been denied life insurance coverage because of her *BRCA1* genotype (Farr 2016). Nor does GINA extend to discrimination in education, lending, housing, or other contexts, though some states have laws that consider these domains (Wood 2017). There have been calls to expand GINA’s coverage (Rothstein 2018). Beyond discrimination, the public’s interest in genetics has driven a market for direct-to-consumer genetic prediction that is almost unregulated in the United States,^5^ including several companies that market aggressively while offering predictions with little or no scientific grounding (Spencer and Topol 2019).

Box 2. “My real résumé was in my cells”

Genetic predictions of phenotypic values are often termed genomic estimated breeding values in the agricultural literature and polygenic scores or polygenic risk scores in the human genetics literature. In human genetics, polygenic scoress usually take the form of weighted sums of individual genotypes, with weights derived from effect-size estimates from genome-wide association studies (GWAS), in which the associations between genotypes at genome-wide loci and a phenotype are estimated in a large sample of individuals.

A widely applied method for estimating a polygenic score is clumping and thresholding, in which the goal is to select loci for inclusion in the polygenic score that are not strongly correlated (clumping) and that each pass a threshold for evidence of association with the phenotype, based on a *P*-value (thresholding). Once *m* loci are selected, the polygenic score $\hat{S}$ is calculated as

$$\hat{S}=\sum_{j=1}^{m}X_{j}{\hat{\beta }}_{j},$$

where ${\hat{\beta }}_{j}$ is the GWAS-estimated effect on the phenotype of carrying one of the alleles at locus *j* and *X_j_* is the number of effect alleles carried by the individual at locus *j*. The thresholds for *P-*value and for correlation among sites can be chosen empirically to maximize predictive accuracy (Euesden *et al.* 2015).

Many variations exist, including approaches to model correlation among sites (Vilhjálmsson *et al.* 2015), methods that take individual-level data rather than GWAS summary statistics as input (Zhou *et al.* 2013), and methods that incorporate locus functional annotations (Hu *et al.* 2017). In addition to the uses in agriculture and medicine discussed in the main text, polygenic scores are used for research, for example to explore relationships among traits (Richardson *et al.* 2019). Choi *et al.* (2020) give an accessible primer on polygenic scores, and Ma and Zhou (2021) review the landscape of current approaches to polygenic score estimation.

At the time of its release, some geneticists viewed *Gattaca* as an antigenetics or antiscience film (Kirby 2000), in part because of its implication—laced throughout the plot rather than stated outright—that precise genetic prediction is extremely difficult for many important human traits (Silver 1997). Viewed from the present, *Gattaca* looks prescient in this regard, agreeing in spirit with the results of 25 years of energetic, multidisciplinary research.^6^

## “This child is still you, simply the best of you”

When planning for their second child, Vincent’s parents opt for a genetically assisted birth: a “valid” child. When Vincent’s parents visit a geneticist (Blair Underwood), they are told, “this child is still you, simply the best of you.” This line and others, as well as the image of 4 candidate embryos on a screen in the geneticist’s office, strongly suggest that “genetic assistance” is some form of embryo or gamete selection rather than CRISPR-like editing of parental genetic material.^7^ However, the geneticist’s next line, “You could conceive naturally a thousand times and never get such a result,” suggests that the process is not simple selection from 4 typical embryos. Perhaps the 4 candidates are picked from a much larger set, or sperm and eggs were submitted to an earlier selection process, or, perhaps, genetic assistance entails human guidance of meiosis.

The geneticist details the features available in the candidate embryos for consideration. The parents opt for a boy and confirm their preference for some cosmetic features—hazel eyes, dark hair, fair skin. (The broad smile of the geneticist, a Black man, as he enunciates “fair skin” brings to mind the question of embryo selection for racialized traits.) As the geneticist continues through a list of traits he has “eliminated,” Vincent’s parents appear uncomfortable and protest, saying that although they are comfortable preventing diseases, “we were wondering if it’s good to just leave a few things to chance.” The geneticist replies, “You want to give your child the best possible start. Believe me, we have enough imperfection built in already.”

The conversation mirrors current discussions about the ethics of preimplantation genetic screening and embryo selection. Forms of embryo selection have been available since before *Gattaca* was released, beginning in 1989 (Handyside *et al.* 1990), when embryos were selected to avoid the development of X-chromosome-linked conditions. Embryo selection has been controversial for decades along many dimensions (Botkin 1998; Knoppers *et al.* 2006; Lázaro-Muñoz *et al.* 2021), including the difficulty and accessibility of necessary in vitro fertilization (IVF) procedures, the ethical basis for selecting embryos, effects on relationships between parents and children, and the reliability of selection procedures.

Current controversy is concentrated on so-called polygenic embryo selection, in which embryos are selected for implantation on the basis of their polygenic scores. Polygenic scores, mentioned in the previous section, are predictions of trait values computed from genetic data, typically as a sum of an individual’s genotypes weighted by each genotypes’ association with the trait, measured in an extremely large sample. Polygenic embryo selection services are already being offered to consumers by at least 4 companies (Turley *et al.* 2021), and the first child born via polygenic embryo selection was born in 2020 (Goldberg 2021).

Several recent studies include models of the performance of embryo selection as a function of the number of embryos available and the predictive accuracy of the polygenic scores (Karavani *et al.* 2019; Lencz *et al.* 2021; Turley *et al.* 2021). In their modeling framework, Karavani and colleagues (2019) find that with modern polygenic scores (assuming their predictive power holds across generations), the expected gain for continuous traits is fairly modest. For example, simulating selection of the embryo with the largest polygenic score from 10 candidates—a large number by current standards—they predict an expected gain of ≈2.5 cm in height.^8^ If multiple traits are targeted for embryo selection, the expected gain per trait can decrease substantially.^9^

For a binary disease, under a liability-threshold model (see Box 1) the expected relative risk reduction can be somewhat more impressive (Lencz *et al.* 2021; This is in part because a considerable fraction of people beyond the disease threshold are only slightly beyond it, meaning that relatively modest shifts in liability may push such an individual from the “affected” to the “unaffected” side.). At the same time, because many complex diseases are fairly rare, relatively impressive improvements in relative risk might entail only minor decreases in absolute risk for a given disease. The decrease in absolute risk may be larger for couples with family history of the disease, and for couples who carry known pathogenic variants, the effects of screening for those variants and polygenic score may stack, leading to more substantial decreases in risk (Kumar *et al.* 2022).

The expected “gain” in a trait value produced by embryo selection is limited by the number of embryos available for selection, which may be low in many IVF settings. At the same time, the rate at which the expected gain increases with the number of embryos is very slow (Box 3). The other important limiting factor is prediction accuracy, which is modest now for most traits, limited in principle by the environmental effects on the trait, and limited further in the near term by the SNP heritability (see previous section).

Turley and colleagues (2021) emphasize difficulties in polygenic embryo selection, many of which could still apply in a *Gattaca*-like world, in which predictive performance might be better than it is now. First, the correlation between polygenic scores in contemporary adults and their trait values is not necessarily a trustworthy estimator of the correlation between embryos’ genotypes and their eventual trait values: for example, if the environment changes over time, the relationship between the polygenic score and the trait value may also change. Furthermore, gene–environment correlations in the population may be responsible for some of the predictive accuracy of polygenic scores. Those correlations are not guaranteed to take the same form within sets of siblings as they do in the general population, which may lead to decrements in accuracy for the relevant prediction problem. (Comparisons among siblings are the relevant scenario for evaluating embryo selection, since embryo selection is typically selection among a candidate set of prospective biological siblings.)

Another issue pointed out by Turley and colleagues is that of genetic correlation or pleiotropy, in which selection for one trait may influence a second trait, perhaps in part because the traits’ variation has partially shared genetic bases, or because one trait is a causal influence on the other for nongenetic reasons. Turley and colleagues give an example in which selecting for educational attainment may simultaneously increase risk for bipolar disorder. Some have argued that this potential issue is minor because genetic correlations among many diseases appear to be mainly positive, such that selecting an embryo with lower heart disease risk (say) would tend to lower risk for several other diseases (Widen *et al.* 2022). At the same time, most current genetic correlation estimates are based on population samples and could be biased by assortative mating (Border *et al.* 2022), or by population stratification or other sources of gene–environment correlation (van Rheenen *et al.* 2019). Within-sibling estimates of genetic correlation are more germane to the embryo selection setting.

Box 3. “You could conceive naturally a thousand times and never get such a result”

Assuming that genetic assistance in *Gattaca* takes the form of embryo selection from a large number of embryos, what might the results be? How different from a couple’s average child (produced the old-fashioned way) might we expect the “assisted” child to be? Several recent papers have taken up the related question of how embryo selection on the basis of polygenic scores might perform (Karavani *et al.* 2019; Lencz *et al.* 2021; Turley *et al.* 2021). A key result from Karavani *et al.* (2019, Equation 1) states that the expected increase (“gain”) in an offspring’s value for a quantitative trait due to being selected as having the largest polygenic score in a set of *n* embryos from the same set of parents approximately obeys

$$E(\mathrm{gain})\propto \sigma_{Z}r_{ps}\sqrt{\;\text{log}\;\;n},$$

where *σ_Z_* is the standard deviation of the trait, and *r_ps_* is the correlation between the polygenic score in the embryo and the eventual trait value of the adult.

The hypothetical that the geneticist in *Gattaca* presents—that of having a thousand children—is accessible in Karavani and colleagues’ expression via the term $\sqrt{\;\text{log}\;\;n}$, arising as an approximation to the expectation of the Gumbel distribution, which is itself an approximation of the distribution of the maximum of a set of *n* independent draws from a normal distribution, with the approximation improving for large *n*. The $\sqrt{\;\text{log}\;\;n}$ term implies that the expected gain that results from selecting the embryo with the highest polygenic score out of 1,000 candidates is only ≈1.7 times as large as that obtained by selecting from 10 candidate embryos.^a^ In other words, the expected gain climbs very slowly with increasing numbers of candidate embryos. That said, increasing the number of offspring does decrease the variance of the gain.

^a^ A more accurate but less intuitive approximation, based on an expression due to Bulmer (1980, Equations 9.11 and 9.12), is $E(\mathrm{gain})\approx \sigma_{Z}r_{ps}\phi (\Phi^{-1}(1-1/n))/\tilde{p}$, with $\tilde{p}=3n/(2n^{2}+1)$, ϕ the probability density function of the standard normal distribution, and Φ the cumulative distribution function of the standard normal (see also Walsh and Lynch 2018, Equations 14.3a and 14.4c). Using this approximation gives a gain from 1,000 candidates approximately ≈2.1 times as large as that from 10 candidates.

The traits targeted by embryo selection are another source of controversy. In *Gattaca*, selection appears to be guided by a discriminatory pseudo-medical consensus about what is desirable. As suggested by Vincent’s parents in *Gattaca*, many people seem more comfortable, in principle, with selection against diseases than with selection for cosmetic or behavioral traits, leaving aside the issue of prediction accuracy (Krones *et al.* 2005; Hudson 2006). Indeed, one of the least controversial cases of embryo screening is for Mendelian conditions lethal in early childhood, like Tay-Sachs disease. The category of “disease” is not always straightforward, however, with, in many cases, social forces and sometimes arbitrary cutoffs determining what counts as a disease (Temple *et al.* 2001). And, of course, the presence of disease, in the abstract, does not determine whether one life is more or less worth living than another.

When reflecting on the limits of current embryo selection, one might also consider how future technology might overcome them. For example, some have pointed to opportunities for gene editing (as opposed to embryo selection), rendering moot the need for parents to carry the desired alleles (Goldstein 2022). But while future technologies may quell some contemporary concerns, others will persist or even become amplified. For example, gene editing will face vexing scientific and ethical questions regarding which alleles to modify, as well as risks from even small error rates, which may well render gene editing counterproductive for its stated goals.

*Gattaca*’s original ending, cut for the theatrical release but included in an advance screening for scientists (Kirby 2000), raised this point with a montage of people who might never have been born if embryo selection against genetic disease had been developed sooner: “Abraham Lincoln (Marfan’s syndrome), Emily Dickinson (Manic Depression), Vincent Van Gogh (Epilepsy), Albert Einstein (Dyslexia), John F. Kennedy (Addison’s Disease), Rita Hayworth (Alzheimer’s Disease), Ray Charles (Primary Glaucoma), Stephen Hawking (Amyotrophic Lateral Sclerosis), and Jackie Joyner-Kersee (Asthma).”

## “We found our man”

Much of the tension in *Gattaca*’s plot is driven by a murder, the unexplained killing of a mission director at the titular facility. The murder gives the film an opportunity to explore genetically assisted law enforcement. The contrast between old and new methods of detection is embodied—initially in reverse—by 2 investigators assigned to the case, an older detective (Alan Arkin) straight out of *noir*, complete with fedora and trenchcoat, and his younger, valid colleague (Loren Dean), who outranks him. (The older detective was presumably born before the era of widespread genetic assistance, and is thus in-valid.)

Vincent is not the murderer, but the investigation nonetheless threatens to uncover his genetic impersonation of Jerome. Though Vincent is fastidious in his effort to keep his identity secret, he nonetheless sheds an eyelash near the crime scene, which ultimately alerts police to the presence of an in-valid in the facility.

Vincent’s illicit presence makes him a prime suspect, leading to a genetic manhunt. When Vincent’s eyelash is analyzed, detectives learn he is an “unregistered” in-valid—i.e. one who is not included in an implied central database—and they see a picture of Vincent, complete with his preimpersonation hairstyle and eyeglasses. They do not receive a name, but they do learn that he used to work as a janitor at the facility, perhaps by questioning his former supervisor (Ernest Borgnine).

Although his picture is known to authorities, Vincent’s appearance does not give him away. Vincent faces greater risk from widespread genetic screening. Detectives appear to test hundreds or even thousands of people while investigating the murder, looking for a match with the stray eyelash. The practice of searching for direct matches between a crime-scene sample and a suspect or database entry was ramping up in the United States around the time of *Gattaca*’s release, following its origin in the United Kingdom in the mid-1980s (Gill *et al.* 1985). Today, the US National DNA Index System (NDIS) contains over 20 million profiles and has been used in over 500,000 investigations (FBI 2021). For each person in the database, genotypes are recorded for either 13 (prior to 2017) or 20 microsatellite loci, known as the CODIS (Combined DNA Index System) markers, along with Y chromosomal and mitochondrial genotypes that are searched in more restricted circumstances. Though the NDIS database is large, many people are not included—inclusion typically depends on prior encounters with law enforcement, though the specific rules vary by state. The current rules have created a database marked by racial disparities in inclusion (Murphy and Tong 2019). Among other issues, racial disparities in the current databases have led to calls for universal databases (Dedrickson 2017; Hazel *et al.* 2018), which are controversial for other reasons, including privacy concerns (Joly *et al.* 2019). Public opinion surveys on universal DNA databases are sparse but do not suggest widespread support for universal databases in the United States or Europe (Dundes 2001; Zieger and Utz 2015).

Despite ambivalence about universal DNA databases, the recent adoption of investigative genetic genealogy (IGG, also called long-range familial search or forensic genetic genealogy) has opened large segments of the population to de facto genetic search. In an IGG search, genetic genealogists upload genome-wide genotypes—not the handful of markers included in CODIS—to platforms typically used for recreational genealogy.^10^ Examining matching segments of DNA likely to be inherited from recent shared ancestors (i.e. identical-by-descent segments), genealogists identify likely biological relatives of the person they seek, aiming to build a family tree that points to their target (Kennett 2019; Katsanis 2020; Kling *et al.* 2021). IGG is powerful because genome-wide markers allow reliable identification of biological relatives out to the third-cousin range, and with some success extending out to fifth cousins or further (Donnelly 1983; Huff *et al.* 2011). Because the typical person has many distant cousins, even a database that includes only a small fraction of the population (≈2%) will include genetically detectable cousins of the majority of the population (Erlich *et al.* 2018; Edge and Coop 2019), potentially allowing most people to be identified by IGG. IGG’s power has caused it to be quickly adopted, and it has produced suspects or identified remains in ∼300 cases (Dowdeswell 2022). At the same time, current regulation (Ram *et al.* 2021) and scientific understanding of IGG are limited, and some of the upload-based databases used for IGG have been flagged for privacy concerns (Edge and Coop 2020; Ney *et al.* 2020).

Speaking of universal databases, the genetic investigation in *Gattaca* raises a puzzle: If Vincent is unregistered, how do the detectives get his picture? And further, how do they have access to his picture but no name attached? One possibility is that the film wants us to assume that the detectives predict his phenotype, generating the picture from the genetic information in the eyelash. The detectives do make reference to other phenotype predictions, including Vincent’s short predicted lifespan and a “violent temperament,” which, in the eyes of the older detective, replaces the need for a plausible motive.

Phenotype predictions in forensic contexts, termed forensic DNA phenotyping, have been explored in the academic literature (Walsh *et al.* 2013; Claes *et al.* 2014; Kayser 2015; Chaitanya *et al.* 2018; Schneider *et al.* 2019) and are currently marketed to law enforcement (Wienroth 2020). Forensic interest in phenotype prediction focuses on so-called externally visible traits (EVTs), which might be perceived by a witness (Jobling 2022). Sex, predicted from sex chromosome karyotype, has been used in this way for some time (Sullivan *et al.* 1993), notwithstanding some errors resulting from imperfect matches between sex chromosome karyotype and sex, or between sex and gender identity or presentation. Other EVTs of interest relate mainly to facial appearance. Although coarse pigmentation prediction (e.g. classifying blue vs brown eyes, but not green vs hazel) can be reasonably accurate for eyes, hair, and skin, at least in some groups (excluding possibilities such as dyed hair, colored contact lenses, graying of hair with age, etc.), predictive performance on most facial traits is not accurate enough for reliable face reconstruction (Schneider *et al.* 2019; Jobling 2022). For some facial phenotypes, predictive accuracy is limited in principle by moderate heritability under current conditions (Šešelj *et al.* 2015; Cole *et al.* 2017; Tsagkrasoulis *et al.* 2017). Furthermore, modifications to one’s appearance (makeup, eyeglasses, jewelry, hairstyle, facial hair style, etc.), though they may strongly influence a person’s appearance, will never be reliably predicted by an individual’s genotype. The facial traits most accurately predicted—pigmentation features—are racialized, and concerns have been raised that predictive phenotyping in forensics contributes to racial profiling (Pollack 2015; Queirós 2019; Hopman and M’charek 2020; Bartram *et al.* 2021).

*Gattaca*’s use of phenotype prediction specifically to investigate a crime is more than a plot device used to explore the ways in which forensic genetics would work in this fictional world. Debates about innateness have been central in conversations around criminal behavior and its social consequences. For example, the search for criminal “types” based in and recognizable in terms of biology has a long history with deep connections to the eugenics movement (Rafter 1997). Myths about inferiority, especially concerning the poor, immigrants, and Black people in the United States, have influenced the development of crime control policies (Lassiter 2015; Hinton 2017; Muhammad 2019). During the search for the murderer, *Gattaca* shows us officers accosting people in an area where in-valids are known to congregate, bringing to mind racial profiling. The film’s depiction of a genetic criminal investigation resonates with the broader theme of genetic discrimination because criminal justice is a setting in which biological discrimination has appeared in flagrant forms.

## “That piece can only be played with twelve”

*Gattaca* artfully weaves its themes into its characters’ interactions. Irene (Uma Thurman) has several roles: she is Vincent’s love interest and a staff member at the *Gattaca* Corporation who is assigned to assist the murder investigation. The interactions between Vincent and Irene generate windows onto the interpretation of genetic “imperfections” in *Gattaca*’s world. Early in the film, Irene takes a nonconsensual look at Vincent’s genetic identity by taking a hair from his desk drawer to a corner genomic sequencing service. (The hair was Jerome’s, stashed by Vincent in his desk for just this eventuality.) When Irene confesses her clandestine genetic monitoring, she also shares her insecurity about a heart defect that prevents her from going to space.

One especially critical moment involving Irene takes place on her first date with Vincent. They attend a performance, and we see a pianist playing an impressive serenade. At the end of the concert, Vincent (posing as Jerome) and Irene stand near a poster advertising the event that highlights the pianist’s 12 fingers. Vincent says, “One finger or twelve, it’s how you play,” to which Irene responds “that piece can only be played with twelve”.^11^ Vincent is speaking from his own experience, suggesting that what one is born with is not what dictates one’s ability. Irene, alternatively, pushes the *Gattaca*-world doctrine of biological determinism—some people are born with the equipment to play certain tunes.

The film is not clear about whether the 12-fingered pianist was the product of genetic assistance (as in, was selected to have 12 fingers to play the piano) or was a “God child” from a natural birth. Whatever the mechanism, the 12-fingered pianist was born with some version of polydactyly, in which vertebrates have additional digits on their extremities (more than 5 digits in humans).^12^ The scene introduces a question about how abnormalities are perceived in the world of *Gattaca*: should we interpret the presumptive celebration of the 6-fingered pianist as a symbol of acceptance or of exoticism? Perhaps the scene highlights that certain conditions are tolerated insofar as they entertain and do not threaten the valid class.

In many settings, conversations around abnormalities, impairments, and disability have become more sophisticated, as scientists, scholars, and advocates have challenged scientific fields to rethink normative definitions of fitness, health, and disability (Branch *et al.* 2022). In the poster Vincent considers after the performance, the pianist’s 12 fingers cover his face, suggesting his feeling of shame in *Gattaca*’s world of physical conformity. But they also let him play a transcendent piece of music that few others can manage. One lesson is that fixed notions of impairment are imprecise. This is not only an ethical message but also a technical one: gene-by-environment interactions are a central concept in modern genetics, defined by how the phenotypic manifestation of genotypic information is dependent on context.

## Conclusion: “But then, who knows what he could do, right?”

###  

*Gattaca* is a work of science fiction, and thus its main charge is not to depict science as we know it, but rather, to ask questions using science-adjacent concepts as vehicles. *Gattaca*’s most obvious pattern of scientific inaccuracy is the persistent overstatement by powerful people—healthcare professionals, police, employers—of the predictive potential of genetic information. This inaccuracy is a feature of the film, not a bug, in that it highlights the social force of such overstatements. More broadly, *Gattaca* introduces questions about contemporary applications of genetics to healthcare, embryo selection, forensics, and other areas. *Gattaca* was ahead of its time, an even richer text today than it was in its day.

In 2022, we stand in the midst of debates about the social implications of advancements in genetic science. These include technical and practical challenges (e.g. missing heritability), and broader questions regarding how new technologies (e.g. gene editing via CRISPR) should be used (Greely 2019). And unfortunately, some voices take on a troubling form, echoing historical attempts to use genetics as a justification for the othering of groups based on presumed differences (Graves 2001; Hammonds and Herzig 2009), fueling scientific racism and biological determinism that stymie the progress of modern genetics (Ogbunu 2022).

A recurring problem at the interface of the science and ethics of genetics, participated in by both scientists and users of science, is a kind of giddiness—an unjustified optimism that genetic research will imminently solve the most relevant questions surrounding human nature and society. For example, Charles Davenport once wrote with exhilaration about the potential for tying all sorts of human traits to the Procrustean bed of Mendelism, including “nomadism,” “shiftlessness,” and love of the sea (Kevles 1995, pp. 48–49). Davenport’s flights of fancy were woven into the American eugenics movement, of which he was a leader, and eventually influenced immigration policy. Many forecasts of genetics’ ability to solve large, complex social problems are driven by similar giddiness, saying that any day, our fates will be captured with simple metrics that can be read at birth or even in utero, just like those of the newborn Vincent or his brother, Anton.

The abuses of science in *Gattaca’*s world are based on science advanced beyond our own, but they mirror ones in our own history. Reflecting on *Gattaca* implores us to look ahead, toward creating a world different from the one it portrays, where science is no longer misused to reinforce social inequality, to limit possibilities, and to transform hopes into dreams deferred.

Supplemental material is available at GENETICS online.

## Supplementary Material

iyac142_Supplementary_Data

## Footnotes

1 Jerome’s middle name, by which he is known for much of the film, is Eugene–this nod to eugenics is one of several winking pointers to the film’s subject matter, along with a prominent helical staircase and the film’s title, composed of the G, A, T, and C of DNA sequence.

2 The movie says he’s outlived his predicted lifespan by “10,000” heartbeats, which is good poetry but amounts to a few hours.

3 We do see evidence that Vincent may have a heart condition, in the form of an apparent tachycardic response to exercise, briefly depicted in an electrocardiogram readout. A cardiologist friend, presented with a screenshot of the ECG, told us, “It looks like a supraventricular tachycardia… These types of rhythms are more common (up to 0.5% of the population), not very specific for particular cardiomyopathies, and usually more benign … In short, we can’t make a diagnosis from this rhythm alone, and this alone probably wouldn’t kill him, unless associated with another more dangerous arrhythmia or heart condition.”

4 In the dairy cattle case, acceleration in improvement comes primarily from shorter generation time—when selecting bulls to sire the next generation, genetic predictions for milk yield of bulls’ daughters are used in lieu of actually testing the yield of bulls’ progeny, which is time consuming. This approach allows for more generations of selection per unit time.

5 Beyond the FDA’s regulation of health-related predictions from 23andMe (Annas and Elias 2014).

6 Limits on the precision of individual trait prediction would have been clear to some geneticists before 1997, including those familiar with estimated heritabilities across traits and species (Mousseau and Roff 1987).

7 In a deleted scene, the geneticist offers to perform additional editing for a price, “Now, you appreciate I can only work with the raw material at my disposal. But, for a little extra, I can also attempt to insert sequences associated with enhanced mathematical or musical ability.” The parents express interest but balk at the high price.

8
Karavani and colleagues (2019) assumed a height predictor that explains 25% of the variance in height. Updating their analysis to use the height prediction accuracy obtained in Yengo *et al.* (2022) meta-analysis for people of European ancestries would revise their estimate up to $2.5\sqrt{.4/.25}\approx 3.2\;cm$.

9 For *T* genetically uncorrelated traits weighted equally, the model of Karavani and colleagues predicts the expected gain per trait will decrease by a factor of $1/\sqrt{T}$ compared with selection on a single trait.

10 Although there is an underappreciated amount of personal information that can be estimated from CODIS genotypes (Algee-Hewitt *et al.* 2016; Edge *et al.* 2017; Kim *et al.* 2018; Bañuelos *et al.* 2021), use of genome-wide genotypes in law enforcement raises further privacy questions.

11
*Gattaca* composer Michael Nyman based the serenade on Schubert’s Impromptu No. 3 in *G_b_* major, adding extra notes that would be impossible for a 10-fingered pianist to play. Asked on Twitter by one of us whether the piece might be playable by a 12-fingered pianist, Mr. Nyman expressed uncertainty, replying with only a shrug emoji, “” (Nyman, personal communication).

12 Fans of *Gattaca* have noted that the 12-fingered piano player may have an uncommon manifestation of polydactyly, as the player appears to have dexterous control over all 12 fingers. Such cases are not typical, but the disease is defined by heterogeneity in presentation from case to case (Malik 2014), and some people with polydactyly have extra digits that can be moved independently, facilitating actions that 5-fingered people cannot perform (Mehring *et al.* 2019).
