# Supplementary material for: Gattaca as a lens on contemporary genetics: marking 25 years into the film’s “not-too-distant” future
Source: Genetics. 2022 Oct 11;222(4):iyac142. doi: 10.1093/genetics/iyac142 (PMC9713434; doi:10.1093/genetics/iyac142)
Supplement: iyac142_Supplementary_Data [file iyac142_supplementary_data.pdf]

Supplementary Information for *Gattaca* as a lens on contemporary genetics: Marking 25 years into the film's "not-too-distant" future

C. Brandon Ogbunugafor[1,2,3†*] and Michael D. Edge[4†*]

[1]Department of Ecology and Evolutionary Biology, Yale University, New Haven, CT 06520 USA
[2]Santa Fe Institute, Santa Fe, NM, 87501 USA
[3]Vermont Complex Systems Center, Burlington, VT, 05401 USA
[4]Department of Quantitative and Computational Biology, University of Southern California, Los Angeles, CA, 90089 USA
[†]These authors contributed equally to this work.

*Corresponding authors: Department of Ecology and Evolutionary Biology, Yale University, New Haven, CT 06520 USA. E-mail: brandon.ogbunu@yale.edu; Department of Quantitative and Computational Biology, University of Southern California, Los Angeles, CA, 90089 USA. Email: edgem@usc.edu

This document contains supplementary text and tables for "*Gattaca* as a lens on contemporary genetics: Marking 25 years into the film's 'not-too-distant' future" by Ogbunugafor & Edge. The first section contains thought questions for group discussions of *Gattaca*, followed by details on the liability threshold model and a derivation o equation 1 from the main text, followed subsequently by Tables S1 and S2.

Thought questions for discussions of Gattaca

Gattaca has been used in educational settings to introduce concepts in genetics and to explore controversies at the intersection between science and society.

In this section, we offer discussion questions that can be used to drive conversations in group settings, based on topics that appear in the film and in modern discourse around genomic technology. This is not an exhaustive list but rather is intended to generate starting points for discussion. There is no sole "correct" answer to any of these questions. We make a rough division into questions about (a) ethical, legal, and social issues; (b) the science of human genetics; and (c) the film and its world. At the same time, many of these questions could be grouped in multiple categories.

A warning for those who haven't seen the film: Major spoilers are embedded in a few of these questions.

Ethical, Legal, and Social Issues

1. *Gattaca* shows a world in which widespread embryo selection becomes the basis for discrimination, despite laws against it. Do you think it is possible to design a society that used widespread embryo selection, as in *Gattaca*, and at the same time to avoid genetic discrimination? Why or why not? How would you attempt to do it?

2. When (if ever), in your opinion, should human embryo selection (or direct modification of genetic material (Greely, 2019)) be allowed? Would you place restrictions on the traits that are allowed as targets of selection or modification? Which traits would you allow? Would you place restrictions on the basis of scientific understanding of the traits or their genetic basis? What kinds of knowledge would you require?

3. Following on the previous question, many people feel that there are certain traits that should not be allowed to be targets of embryo selection. However, as discussed briefly in the main text, the existence of pleiotropy means that selection on a target trait could have an effect on other traits. Given pleiotropy, what does it mean to specify a set of traits that are not allowed as (direct) targets of embryo selection?

4. The film suggests that a world can exist whereby discrimination is no longer based on the "color of your skin" (race and ethnicity), but rather, on the composition of one's genome. Do you think such a world, where one form of discrimination (genetic discrimination) fully supplants another (racial discrimination) is possible? Why or why not? Are there historical analogues for this happening?

5. As mentioned in the main text, *Gattaca*'s original ending included montage of people who might never

have been born if embryo selection against genetic disease had been developed sooner. It also suggested that one person who might not have been born is "you." What do you make of of these statements as arguments?

6. In the *Gattaca* detectives' search for the murderer, they collect DNA samples from hundreds of people on the basis of only their location (being either Gattaca employees at the facility, people congregated in a place where in-valids are thought to assemble, and people within a radius of the crime scene). This kind of procedure is sometimes called a "DNA dragnet" and has been carried out in our world, including around the time *Gattaca* was made. What are some of the risks or ethical costs of conducting a DNA dragnet? How effective would you expect DNA dragnets to be in identifying people of interest? Why?

7. When, if ever, do you think genetic prediction of a suspect's phenotypes ought to be allowed in criminal investigations? What kinds of phenotypes should be allowed, and what level of accuracy and precision in prediction should be required? In which kinds of cases would you authorize forensic DNA phenotyping?

8. The DNA from Vincent's stray eyelash is compared against what seems to be a near-universal database of in-valids. (Vincent's feat of remaining unregistered is noted as an indicator of his cleverness.) What, in your opinion, are the costs and benefits of maintaining a universal national DNA database, in which every resident of a nation would have a "DNA fingerprint" stored? Would you favor such a database in your country? Why or why not?

9. *Gattaca* portrays or suggests genetic discrimination in many contexts: hiring, promotion, education, health insurance coverage, criminal investigation, and even dating. Are there other contexts in which you think genetic discrimination could become a problem? What are they?

Genetics

10. Vincent says that most of the conditions he is predicted to develop at birth are "still untreatable to this day." Is the fact that treatment is impossible for these conditions a warning sign about the accuracy of the predictions themselves? Why or why not? More generally, what are the gaps between the ability to predict an outcome and the ability to intervene in it downstream?

11. A trait's heritability is not a fixed property of the trait; it can vary on the basis of the genetic variation in the population and on the amount of environmental variation that influences the trait. In the world of *Gattaca*, how would you expect traits' heritabilities to change? Specifically, how do you think that the components of heritability, traits' genetic variance and environmental variance, would change? More generally, what would be the long-term effects on the population of embryo selection as practiced in *Gattaca*?

12. Turley and colleagues (2021) raise the point that prediction of an embryo's traits is dependent on the environment that the embryo experiences. The embryo's environment might be different from previous environments, which can be the only basis for prediction. For what kinds of traits do you think that this disconnect will be most relevant, and most likely to lead to prediction errors?

13. As mentioned in the main text, the first child born after polygenic embryo selection was born in 2020. The first report of children born from gene-edited embryos came even earlier, in 2018, after a secret experiment led by He Jiankui that was roundly condemned by scientists and biomedical ethicists (Greely, 2019). Discuss the difference between the risks and benefits represented by embryo selection procedures, including polygenic embryo selection, and direct genetic editing of embryos.

14. [For groups familiar with human genetics study designs] As mentioned in the main text, the SNP heritability is likely the near-term upper bound on the percentage of a trait's variance that could be explained with a genetic predictor. Do you think that genetic predictions' performance will exceed the SNP heritability in the long term? Why or why not? If you said, "yes," how do you think that such predictions could be developed? Will they achieve the performance suggested by twin-based heritability estimates, or will they be bounded by some other number?

Gattaca and its world

15. In a deleted scene, the geneticist offers Vincent's parents an opportunity, for a steep fee, to insert genetic material into one of their embryos, sequences associated with advanced musical or mathematical ability. Why do you think the filmmakers deleted that scene? How would its inclusion change your perception of the *Gattaca* world?

16. Do you think that the world of *Gattaca* is actually free of racial discrimination, as claimed in Vincent's narration? Why or why not? (Consider Vincent's parents' preference for "fair skin" in their second child—what might it mean?)

17. The film has little explicit to say about gender or sex. (*Gattaca* passes only the first of three parts of the Bechdel test, by having two named women characters, Marie [Vincent's mother] and Irene, who do not

speak to each other in the film.) Do you think that such a world can exist without gender or sex bias? How would sexism manifest in a world like that of *Gattaca*? (Consider that Vincent places responsibility for his being conceived as a "God child" solely in the hands of his mother. Why do you think he says this, and what might it mean about the world of *Gattaca*?)

18. Do you think that the 12-fingered pianist from the film was born to play challenging music (via genetic assistance), or is he instead a "God child"? Explain your answer.

19. Some have suggested that Vincent is a villainous character in *Gattaca*, in that his goal of becoming an astronaut would put his crewmates at risk because he would likely die in space. Does this reading of the film resonate with you? Why or why not?

20. The film ends with Vincent achieving his lifelong goal of reaching the stars, and Jerome committing suicide. Why do you think Jerome commits suicide, and what does it say about the world of *Gattaca*, and about Jerome himself?

21. Do you think that Vincent returns from his trip, or does his purported heart condition (or something else) kill him while he is on his mission? Why do you think so, or think not?

22. Assuming Vincent returns home safely, what do you think happens to him after his arrival back on Earth? Now that his life dream has been achieved, do you think he continues the "borrowed ladder" lifestyle?

23. If it were to become widely known that Vincent, an in-valid, completed astronaut training and qualified for an important mission, what would be the effect in the world of *Gattaca*?

Literature cited

Greely HT. 2019. CRISPR'd babies: human germline genome editing in the 'he jiankui affair'*. Journal of Law and the Biosciences. 6:111–183.

Turley P, Meyer MN, Wang N, Cesarini D, Hammonds E, Martin AR, Neale BM, Rehm HL, Wilkins-Haug L, Benjamin DJ *et al.* 2021. Problems with using polygenic scores to select embryos. New England Journal of Medicine. 385:78–86.

Details on the liability threshold model and derivation of equation 1

Under the liability threshold model, an individual's liability is represented as a normally distributed random variable, itself a sum of two independent normally distributed variables, one representing a "genetic" component of liability, and the other representing an "environmental" component of liability. The individual develops the disease if their total liability exceeds a threshold. We do not observe an individual's liability, only whether the individual exceeds the threshold and thus develops the disease. The liability threshold model is a coarse statistical description of complex disease risk. That said, many of its basic predictions approximately hold for many complex diseases in humans (Visscher and Wray 2015).

To model a specific disease under the liability threshold model, one must specify a disease prevalence, which determines the threshold, and a heritability, which determines the relative variance of the genetic and environmental components of liability. For example, a rare disease will have a high threshold, such that only a few people have liabilities exceeding the threshold. For a highly heritable disease, the variance of the genetic component of liability will be higher than that of the environmental component of liability, such that the genetic liability is responsible for most of the variance in overall liability. One way to write the model is

$$P(D) = P(L_G + L_E > t) \quad (1)$$

where D represents the event that the individual develops the disease, L_G is the genetic component of liability, L_E is the environmental component of liability, and t is a threshold. In one of several possible parameterizations, L_G and L_E are both normal random variables with expectation 0 and variances σ_G^2 and σ_E^2, with $\sigma_G^2 + \sigma_E^2 = 1$, so that the liability is a standard normal. In this parametrization, σ_G^2 is the heritability of the liability. The threshold is then chosen as $t = \Phi^{-1}(1-p)$, where p is the disease prevalence and Φ is the cumulative distribution function of the standard normal distribution.

Under this model, define r an individual's predicted risk for the disease. We seek the quantile of the genetic liability necessary to obtain disease risk r given prevalence p and liability-scale heritability σ_G^2.

If an individual has genetic risk r, then by definition, $P(L_G + L_E > t | L_G = l_G) = r$, i.e. the probability their total liability exceeds the threshold, conditional on their realized genetic liability, l_G, is r. This statement implies $P(L_E > t - l_G) = r$, which is equivalent to $P(L_E \leq t - l_G) = 1 - r$. The left side is the cumulative distribution function of L_E evaluated at $t - l_G$, so by inverting the cumulative distribution at $1 - r$ (that is, by calling the quantile function of L_E at $1 - r$), we obtain $t - l_G$. If Φ^{-1} is the quantile function of the standard normal, then $\sigma_E \Phi^{-1}$ is the quantile function of a normal distribution with expectation 0 and standard deviation σ_E, so we have

$$t - l_G = \sigma_E \Phi^{-1}(1-r).$$

And, remembering that $t = \Phi^{-1}(1-p)$, we have that the implied genetic liability is

$$l_G = t - (t - l_G) = \Phi^{-1}(1-p) - \sigma_E \Phi^{-1}(1-r).$$

Ultimately, we seek the quantile associated with risk r, and thus genetic liability l_G, obtained by evaluating the cumulative distribution function of L_G at l_G. L_G is normal with expectation 0 and standard deviation σ_G, so its cumulative distribution function can be obtained by dividing the argument by σ_G and using the standard normal cumulative distribution function. Thus, the percentile of the genetic liability L_G necessary to produce risk r is

$$\Phi(l_G/\sigma_G) = \Phi([\Phi^{-1}(1-p) - \sigma_E \Phi^{-1}(1-r)]/\sigma_G),$$

which is equal to equation 2. The relationship of individual risk percentage and individual risk quantile considered here is also the subject of the "predictiveness curve" considered by So & Sham (2010).

Literature cited

So HC, Sham PC. 2010. A unifying framework for evaluating the predictive power of genetic variants based on the level of heritability explained. PLOS Genetics. 6:1–13.

Visscher PM, Wray NR. 2015. Concepts and misconceptions about the polygenic additive model applied to disease. Human Heredity. 80:165–170.

Table S1 Definitions of key concepts and terms

Term	Definition
"Valid" and "in-valid"	Terms from *Gattaca* that refer to people born either with ("valid") or without ("in-valid") genetic assistance. We use these terms because they are the most frequent in the film, but valids are also referred to as vitros or "made men," and in-valids as God-children, faith births, and uteros.
"Borrowed ladder"	In the world of *Gattaca*, the illegal act of adopting another individual's genetic identity in order to gain access to exclusive parts of society (reserved for the "valid"). People who attempt such impersonation are also called "de-gene-rates."
Genetic prediction	The attempt to predict an organism's phenotype from its genotype.
Heritability	The proportion of a trait's variance attributable to genetic variation in a given population. A trait's heritability is not fixed and depends on both genetic variation and environmental conditions. The precision of genetic prediction is limited by the heritability. The heritability explained by common variants is called the "SNP heritability."
Genetic interactions	Encompassing gene-by-gene interactions and gene-by-environment interactions. Interaction describes a situation in which the effect of a genotype on an individual outcome depends on either another genotype (gene-by-gene interaction) or the organism's environment (gene-by-environment interaction).
Polygenic score	A prediction of a phenotype formed from an individual's genotype, in practice typically a weighted sum of a person's counts of alleles associated with the phenotype. Also called a polygenic risk score (PRS).
Embryo selection	The selection of a candidate embryo produced by in vitro fertilization for implantation on the basis of its predicted phenotype. Also called embryo screening or preimplantation genetic screening. Though some have taken *Gattaca* to be a movie about gene editing, the film suggests that the key technology of "genetic assistance" is embryo selection.
DNA "fingerprint"	A subset of a person's genotype, typically from a small number of highly variable markers, chosen so that two unrelated people are extremely unlikely to share genotypes at all markers. Heavily used in forensic genetics.
Investigative genetic genealogy (IGG)	The attempt to identify the source of a crime-scene sample by identifying people who appear to be biological relatives of the source in genetic genealogy databases.
Forensic DNA phenotyping	A special case of genetic prediction, in which DNA is used to predict externally visible phenotypes for forensic purposes.
Polydactyly	A condition in which an organism develops additional digits on its extremities. *Gattaca* features a pianist with six fingers per hand.

Table S2 Some important characters in *Gattaca*

Character	Description	Actor
Vincent Anton Freeman / Vincent-Jerome (adult)	The main character. Vincent, an "in-valid" or "god child' born without the aid of genetic technology, genetically impersonates a "valid," Jerome, in order to become an astronaut.	Ethan Hawke
Jerome Eugene Morrow	The person from whom Vincent adopts his genetic identity. A valid with an exceptional genetic profile. A former competitive swimmer who has fallen on hard times.	Jude Law
Anton Freeman	Vincent's younger brother. Unlike Vincent, Anton is conceived with the aid of genetic technology.	William Lee Scott
Irene	A staff member at the Gattaca Corporation. A valid whose risk for a heart condition nonetheless prevents her from going to space. Vincent's love interest.	Uma Thurman
Geneticist	Oversees Anton's conception using genomic technology, and explains to the family the benefits of such technology.	Blair Underwood
